# Generation of Rh D‐negative blood using CRISPR/Cas9

**DOI:** 10.1111/cpr.13486

**Published:** 2023-04-25

**Authors:** Lei Xu, Quan Zeng, Liqing Liang, Zhou Yang, Mingyi Qu, Huilin Li, Bowen Zhang, Jing Zhang, Xin Yuan, Lin Chen, Zeng Fan, Lijuan He, Xue Nan, Wen Yue, Xiaoyan Xie, Xuetao Pei

**Affiliations:** ^1^ Stem Cell and Regenerative Medicine Lab Beijing Institute of Radiation Medicine Beijing China; ^2^ South China Research Center for Stem Cell & Regenerative Medicine Guangzhou China; ^3^ Institute of Health Service and Transfusion Medicine Beijing China

## Abstract

Blood supply shortages, especially the shortage of rare blood types, threaten the current medical system. Research on stem cells has shed light on in vitro blood cell manufacturing. The in vitro production of universal red blood cells (RBCs) from induced pluripotent stem cells (iPSCs) has become the focus of transfusion medicine. To obtain O‐type Rh D‐negative blood, we developed O‐type Rh D‐negative human (h)iPSCs using homology‐directed repair (HDR)‐based CRISPR/Cas9. HuAiPSCs derived from human umbilical arterial endothelial cells and showing haematopoietic differentiation preferences were selected for gene modification. Guide RNAs (gRNAs) were selected, and a donor template flanked by gRNA‐directed homologous arms was set to introduce a premature stop code to *RHD* exon 2. CRISPR/Cas9 gene editing has resulted in the successful generation of an *RHD* knockout cell line. The HuAiPSC‐A1‐RHD^−/−^ cell line was differentiated into haematopoietic stem/progenitor cells and subsequently into erythrocytes in the oxygen concentration‐optimized differentiation scheme. HuAiPSC‐A1‐RHD^−/−^ derived erythrocytes remained positive for the RBC markers CD71 and CD235a. These erythrocytes did not express D antigen and did not agglutinate in the presence of anti‐Rh D reagents. In conclusion, taking the priority of haematopoietic preference hiPSCs, the HDR‐based CRISPR/Cas9 system and optimizing the erythroid‐lineage differentiation protocol, we first generated O‐type Rh D‐negative universal erythrocytes from *RHD* knockout HuAiPSCs. Its production is highly efficient and shows great potential for clinical applications.

## Graphical abstract

To generate O‐type Rh D‐negative universal RBCs, an hiPSC line, HuAiPSC, which was derived from human umbilical arterial endothelial cells and showed haematopoietic development preference, was genetic modified to knockout *RHD* gene with CRISPR/Cas9. The modified seed cells, HuAiPSC‐A1‐RHD^−/−^ cells were induced towards erythroid cells with optimized protocol, which combined hypoxia and normoxia conditions for better erythrocyte outcome. Consequently, O‐type Rh D‐negative universal RBCs were efficiently generated, holding great potential in clinic applications.

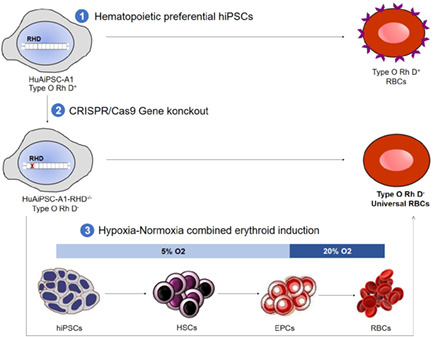

## 1 INTRODUCTION

Blood transfusion is an important therapeutic approach for emergency transfusion applications as well as for patients with severe anaemia.^1^, ^2^ Although many blood banks exist globally, factors such as natural disasters, accidents and unpredictable pandemics have resulted in blood shortages.^3^ In 2019, the global coronavirus of 2019 (COVID‐19) pandemic severely affected blood banks worldwide, resulting in a global shortfall in blood supply,^3^ further increasing the pressing need for blood alternatives. Meanwhile, blood group compatibility (ABO and Rh blood type) and the risk of infection can pose significant challenges for blood transfusion.^2^, ^4^ Therefore, alternatives are required for patients with rare blood groups.^5^

O‐type Rh D‐negative blood, the universal donor blood type, is considered a limited and precious source of red blood cells (RBCs) for transfusion applications.^6^, ^7^, ^8^ The unlimited proliferation potential of human induced pluripotent stem cells (hiPSCs) coupled with their differentiation potential into all three germ layer cell types, offers great promise and opportunities for cell‐based therapy and regenerative medicine.^9^ Therefore, the production of RBCs from hiPSCs is of notable clinical practical value because it could become an alternative source of cells for transfusion.^10^ Universal RBCs differentiated from O‐type hiPSCs may overcome the blood shortage in patients with rare blood.^8^

Remarkably, hiPSCs derived from different somatic cells have demonstrated significant differentiation potential towards their parental cells.^11^, ^12^, ^13^, ^14^ Our previous study showed that, based on epigenetic memory, hiPSCs derived from human umbilical arterial endothelial cells (HuAECs) have great priority in the generation of haematopoietic cells compared with hiPSCs derived from human umbilical cord vein endothelial cells, cord blood, human foetal skin fibroblasts or human foreskin fibroblasts.^15^ Therefore, HuAECs‐derived hiPSC, referred to as HuAiPSCs, are potentially ideal origin cells for generating universal RBCs. Because of tissue specificity and a low proportion of Rh D‐negative population, finding an O‐type Rh D‐negative HuAEC donor is a challenge. Fortunately, the breakthrough in hiPSCs combined with gene editing technology has established an opportunity for the generation of universal RBCs.

The current study was undertaken to develop O‐type Rh D‐negative hiPSCs with a high potential for haematopoietic differentiation using gene editing. CRISPR/Cas9 was used to disrupt the *RHD* gene of O‐type HuAiPSCs. Subsequently, O‐type Rh D‐negative HuAiPSCs were induced to differentiate into the desired erythroid lineage. The hiPSC‐RBC induction protocol was optimized using a combination of hypoxic and normoxic culture conditions for efficient RBC generation.^16^, ^17^, ^18^ Using haematopoietic bias hiPSCs, gene modification, and an optimized induction protocol, O‐type Rh D‐negative blood can be efficiently generated.

## 2 MATERIALS AND METHODS

### 2.1 Cell culture

Human iPSC lines HuAiPSC‐A1^19^ and HuAiPSC‐A1‐RHD^−/−^ were cultured in Essential 8 (Gibco, Grand Island, NY) on Matrigel‐coated wells (Corning, New York). Cells were maintained daily and passaged every 4–6 days to maintain undifferentiated growth, as previously described.^19^ When colonies reached 70%–80% confluency, ReLeSR (StemCell Technologies, Vancouver, BC, Canada) was used to detach and dissociate large clones, and cells were passaged at a 1:10–1:20 ratio. Single cells were obtained using Accutase (StemCell Technologies) before plasmid transfection and fluorescence‐activated cell sorting (FACS). A rock inhibitor (Y‐27632 2HCl, Selleck, Houston, TX) was used to improve the cell survival rate during replating. All cultures were maintained at 37°C in a 5% CO_2_ incubator (Thermo Scientific, Waltham, MA).

### 2.2 Guide RNA design

Guide RNAs (gRNA) were designed using online portals (crispr.mit.edu and CCTop the CRISPR/Cas9 target online predictor). Exons that encode key regions of the protein in question were selected. The selected gRNAs were as follows:

Exon 2: 5′‐GCTGTGTCTCCGGAAACTCG‐3′.

Exon 5‐1: 5′‐ACGGCATTCTTCCTTTCGAT‐3′.

Exon 5‐2: 5′‐GCTGACTGCTACAGCATAGT‐3′.

Exon 7‐1: 5′‐GGGCTACAACTTCAGCTTGC‐3′.

Exon 7‐2: 5′‐GCTGAAGTTGTAGCCCATGA‐3′.

Exon 7‐3: 5′‐GATACCGTCGGAGCCGGCAA‐3′.

gRNAs were cloned into the LentiCRISPR v2 vector (ADDGENE, Massachusetts). K562 cells were used to identify successful gRNA combinations for the knockout of the selected genes.

### 2.3 Generation of the *RHD* knockout HuAiPSC‐A1 cell line

To obtain the *RHD* knockout cell line, homologous recombination was used to repair the DNA sequence after gene editing. *RHD*‐specific gRNA was cloned into the Cas9 expression vector (SH100, GeneCopoeia, Maryland) by replacing the original AAVS1 targeting gRNA to generate the pRHD‐Exon 2‐sgRNA‐CRISPR‐Cas9 vector. RHD‐exon 2 left and right homologous arms, containing the sequence of the premature stop code, were cloned into the GFP/puromycin expression vector (SH200, GeneCopoeia) to produce the pRHD‐HL‐stop‐HR‐donor‐puro‐GFP vector (Figure S1). Specifically, homologous fragments were amplified from the genome of the hESC‐H1 cell line and cloned into the pClone007 vector (TSINGKE, Beijing, China). Then, the vector SH200 was digested with HpaI/XhoI (NEB, New Jersey), the AAVS1 right homologous arm was removed, and a HpaI/SalI‐digested RHD‐exon 2 right homologous arm fragment from pClone007‐RHD‐Right was ligated into the donor vector. Subsequently, the RHD‐exon 2 left homologous arm was digested with KpnI/XbaI (NEB) from pClone007‐RHD‐left and inserted into the KpnI/XbaI‐digested donor vector.

Confluent HuAiPSC‐A1 cells (40%–50%) were transfected with 500 ng of CRIPSR/Cas9 plasmids (250 ng pRHD‐exon 2‐sgRNA‐CRISPR‐Cas9 and 250 ng pRHD‐HL‐stop‐HR‐donor‐puro‐GFP) using Lipofectamine Stem Transfection Reagent (Invitrogen, Carlsbad, CA, USA). Untransfected HuAiPSC‐A1 served as the negative control. After incubation for 2 days at 37°C, the cells were treated with 0.5 μg/mL puromycin (InvivoGen, California). After 2 weeks of drug selection, the puromycin‐resistant clones were expanded. GFP‐positive cells were sorted using FACS.

### 2.4 Sequencing analysis

To confirm *RHD* KO colonies, genomic DNA was extracted using the TIANamp Genomic DNA Kit (TIANGEN, Beijing, China) and polymerase chain reaction (PCR) was performed using the Q5® High‐Fidelity 2xMaster Mix (NEB). The primers used are listed in Table S1.

### 2.5 Mycoplasma test

Mycoplasma was detected using a Mycoplasma Detection Set (Macgene, Beijing, China), according to the manufacturer's instructions.

### 2.6 Karyotype and short tandem repeat analyses

Cells were treated with colcemid (Selleck), harvested and treated with hypotonic solution for karyotype analysis, as previously described.^19^ At least 20 metaphases for each sample were analysed with regard to chromosome number and structural rearrangements by the Guangzhou Kingmed Center for Clinical Laboratory. Short tandem repeat (STR) analysis was performed with the detection of 21 loci by Microread Genetics Co. Ltd. (Beijing, China).

### 2.7 Alkaline phosphatase staining

Alkaline phosphatase (AP) activity was measured using an AP staining solution kit (Beyotime, Shanghai, China), according to the manufacturer's instructions.

### 2.8 Embryoid bodies (EBs) differentiation

Confluent (iPSCs) were detached from Matrigel using Accutase (StemCell Technologies) for 3–5 min at 37°C. To form EBs, cells were washed thrice, resuspended in Essential 8 (Gibco) supplemented with ROCK inhibitor (Y27632, 10 μmol/L), and plated at a density of 2 × 10^5^ cells/well in ultra‐low attachment 6‐well plates (Corning). After 24 h, the EBs were harvested and resuspended in Essential 6 medium (Gibco). The medium was changed every alternate day. After 7 days, the cells were transferred into Matrigel‐coated (Corning) wells for adherent growth. The medium was replaced with fresh medium every alternate day. The cells were allowed to differentiate for 7 days for further immunostaining analysis.

### 2.9 Teratoma formation

The mice were manipulated and housed according to protocols approved by the Institutional Animal Care and Use Committee (IACUC) in compliance with the Beijing Medical Experimental Animal Care Commission. (Reference number: IACUC of AMMS‐13‐2016‐016) 5 × 10^6^ cells were resuspended in 80 μL of precooled (4°C) Matrigel (Corning) and maintained on ice until intramuscular injection into NOD/SCID mice. Teratomas are typically formed within 8–10 weeks of injection. Teratomas were dissected, fixed in 4% paraformaldehyde, embedded with paraffin, sectioned, and then stained with haematoxylin and eosin (H&E) and subjected to immunostaining analysis.

### 2.10 Immunofluorescence

For immunofluorescence staining, cells were fixed with 4% paraformaldehyde, permeabilized with 0.2% Triton X‐100, blocked in 3% bovine serum albumin (BSA), and further incubated with primary antibodies in Dulbecco's phosphate‐buffered saline (DPBS) with 3% BSA overnight at 4°C. Cells were then incubated with the corresponding secondary antibodies for 45 min in the dark at room temperature. The cells were counterstained with 4′,6‐diamidino‐2‐phenylindole (1:500 dilution) for 15 min, maintained in the dark at room temperature, and visualized using fluorescence microscopy. The antibodies used are listed in Table S2.

### 2.11 Induction of hiPSC differentiation into erythrocytes

The in vitro generation of erythrocytes comprises four phases: (I) mesoderm induction, (II) hemogenic endothelium (HE) commitment, (III) haematopoietic cell emergence and erythroid differentiation and (IV) erythrocyte maturation. Cells were differentiated into mesoderm and subsequently subjected to HE using a previously published protocol with modifications.^20^, ^21^

Briefly, in Phase I (Days −1 to 2), 70%–80% confluent hiPSCs were treated with Accutase (StemCell Technologies) for 3–5 min in a 37°C incubator. Small, scraped clumps were collected, centrifuged, resuspended in Essential 8 (Gibco) supplemented with 10 μmol/L Y27632 (Selleck) and plated at a density of 2 × 10^5^ cells/well in ultra‐low attachment 6‐well plates (Corning). After 24 h (Day 0), cell aggregates were harvested and resuspended in differentiation medium (DM) 1 consisting of advanced Dulbecco's medium/F12 supplemented with 1% GlutaMAX, 1% penicillin/streptomycin, 50 μg/mL l‐ascorbic acid 2‐phosphate (AA2P), 25 ng/mL BMP4, 25 ng/mL bFGF and 25 ng/mL activin A. The medium was changed daily during this phase.

In phase II (Days 2–6), the EBs were collected and resuspended in DM2, consisting of advanced Dulbecco's medium/F12 supplemented with 1% GlutaMAX, 1% penicillin/streptomycin, 50 μg/mL AA2P, 25 ng/mL bFGF, 50 ng/mL VEGF_165_ and 2 μmol/L SB431542 (TGFβ inhibitor). The medium was changed daily during this phase.

In phase III (Days 6–15), the EBs were collected and resuspended in DM3, consisting of BEL medium^20^, ^22^ (Table S3) supplemented with 50 ng/mL SCF, 20 ng/mL TPO, 20 ng/mL IL‐3, 20 ng/mL Flt3L, 20 ng/mL VEGF_165_, 5 U/mL EPO, 100 μg/mL transferrin and 10 μmol/L SB431542. The medium was changed every 2 days during this phase.

In phase IV (Days 15–18), for further maturation, cells were collected and passed through a 40 μm cell strainer, washed with DPBS, and resuspended in DM4 containing BEL (without BSA) supplemented with 2.5% AB serum, 5 U/mL EPO and 3 U/mL Heparin Solution. Cells were seeded at a density of 5 × 10^6^ cells/well in 6‐well plates to allow non‐erythroid cells to attach.^23^ The medium was half‐changed daily during this phase. The cultures were maintained at 37°C in 5% CO_2_ and a designated concentration of oxygen in a humidified atmosphere according to the schedule.

On Day 18, non‐adherent cells were collected by passing through a 40 μm cell strainer for erythrocyte analysis. Cells were maintained at 37°C in 5% CO_2_ and 20% oxygen in a humidified atmosphere.

Cell number and morphology were assessed using a cell viability analyser (Beckman Counter, California) and Wright‐Giemsa (BASO, Zhuhai, Guangdong, China) staining, respectively. At the end of the cultivation period, we assessed the number of total cells, erythroid cells, CD34^+^ cells, T cells, B cells, natural killer cells, mononuclear cells and neutrophils using the method described below.

The main reagents used for cell culture and differentiation are listed in Table S2.

### 2.12 Quantitative reverse‐transcription PCR analysis

mRNA was extracted from cells using TRIzol reagent (Invitrogen) and reverse transcribed using ReverTra Ace quantitative reverse‐transcription (qRT‐)PCR Master Mix (TOYOBO, Osaka, Japan), according to the manufacturer's instructions. qRT‐PCR was performed using the THUNDERBIRD SYBR qPCR Mix (TOYOBO) on a Bio‐Rad CFX Connect™ (Bio‐Rad, Hercules, CA). The primers used for detection are listed in Table S1.

### 2.13 Flow cytometry and FACS

The cultured cells were harvested on Days −1, 2, 6, 9, 15 and 18, and their immunophenotypes were determined as follows. On Days 2, 6 and 9, the cells were dissociated into single cells using Accutase (StemCell Technologies) with collagenase D (Sigma, St. Louis, MO) and DNase I (Sigma) and then resuspended in MACSima™ Running Buffer (Miltenyi Biotec, Bergish Gladbach, Germany). On days 15 and 18, the cells were directly passed through a 40 μm cell strainer, washed, and resuspended in Hanks' Balanced Salt Solution (HBSS). The cells were incubated with isotypic antibodies or the indicated antibodies for 30 min at 4°C. The cells were then washed and suspended in HBSS for analysis. A total of 10 nM Syto62 (BD Biosciences, San Jose, CA) was added to the corresponding samples, which were incubated for 10–30 min before analysis. The following antibodies were used: PE‐Brachyury (R&D Systems, Aimolivel, California, CA), APC‐KDR (R&D Systems), APC‐SSEA4 (BD Biosciences), APC‐CD34 (BD Biosciences), PE‐CD45 (BD Biosciences), BV421‐CD43 (BD Biosciences), APC‐CD71 (BD Biosciences), BV421‐CD235a (BD Biosciences), FVS510 (BD Biosciences), APC‐CD31 (eBioscience, San Diego, CA), PECY7‐CD71 (eBioscience), APC‐Syto62 (Invitrogen) and PE‐TRA‐1‐60 (MACS).

For intracellular staining of brachyury, the procedure was performed according to the manufacturer's instructions (R&D Systems). For Rh D analysis, the cells were blocked with 10% donkey serum for 1 h at room temperature. The cells were then incubated with Rh D antibody (Biorbyt; dilution1:100) for 1 h at 20°C. Next, the cells were washed with DPBS and stained with donkey anti‐rabbit AF647 (Invitrogen) for 45 min at room temperature. Cells were washed and stained with BV421‐CD235a, PECY7‐CD71 and FVS510 for 30 min at 4°C. Live cells identified by FVS510 exclusion were analysed for surface marker expression using a BD FACSAria II cell analyser (BD Biosciences). The percentages of positive cells were determined and compared with isotype controls, and the analyses were performed using FlowJo software (Version 10; TreeStar, Ashland, OR). The antibodies used are listed in Table S2.

For FACS of immunolabelled cells, a BD FACSAria II Cell Sorter was used to isolate the GFP^+^/SSEA4^+^/TRA‐1‐60^+^ population.

### 2.14 Agglutination assay

Agglutination assay was performed as previously described.^7^ Briefly, differentiated HuAiPSC‐A1 and HuAiPSC‐A1‐RHD^−/−^ cells as well as control RBCs were plated in 96‐well plates at 1 × 10^6^ cells per well in 10 μL of DPBS. Next, 10 μL of anti‐D blood grouping reagent was added to each well and mixed, incubated the mixture for 15 min at 37°C. The incubated cells were observed using a digital camera (Nikon Corporation, Tokyo, Japan).

### 2.15 Functional analysis of haemoglobin

After washing with DPBS, 5 × 10^7^ cells per group were analysed. Oxygen equilibrium curves were determined using an oxygenation‐dissociation analyser (BLOODOX‐2018 Analyzer, Softron Biotechnology, Beijing, China), as previously described.^24^ Human adult peripheral blood cells were used as controls.

### 2.16 Statistical analysis

All data are expressed as the mean ± SD. Statistical significance was determined using a two‐tailed Student's *t*‐test. A *p*‐value <0.05 was considered statistically significant.

## 3 RESULTS

### 3.1 Design and validation of gRNAs targeting *RHD*

**FIGURE 1:**
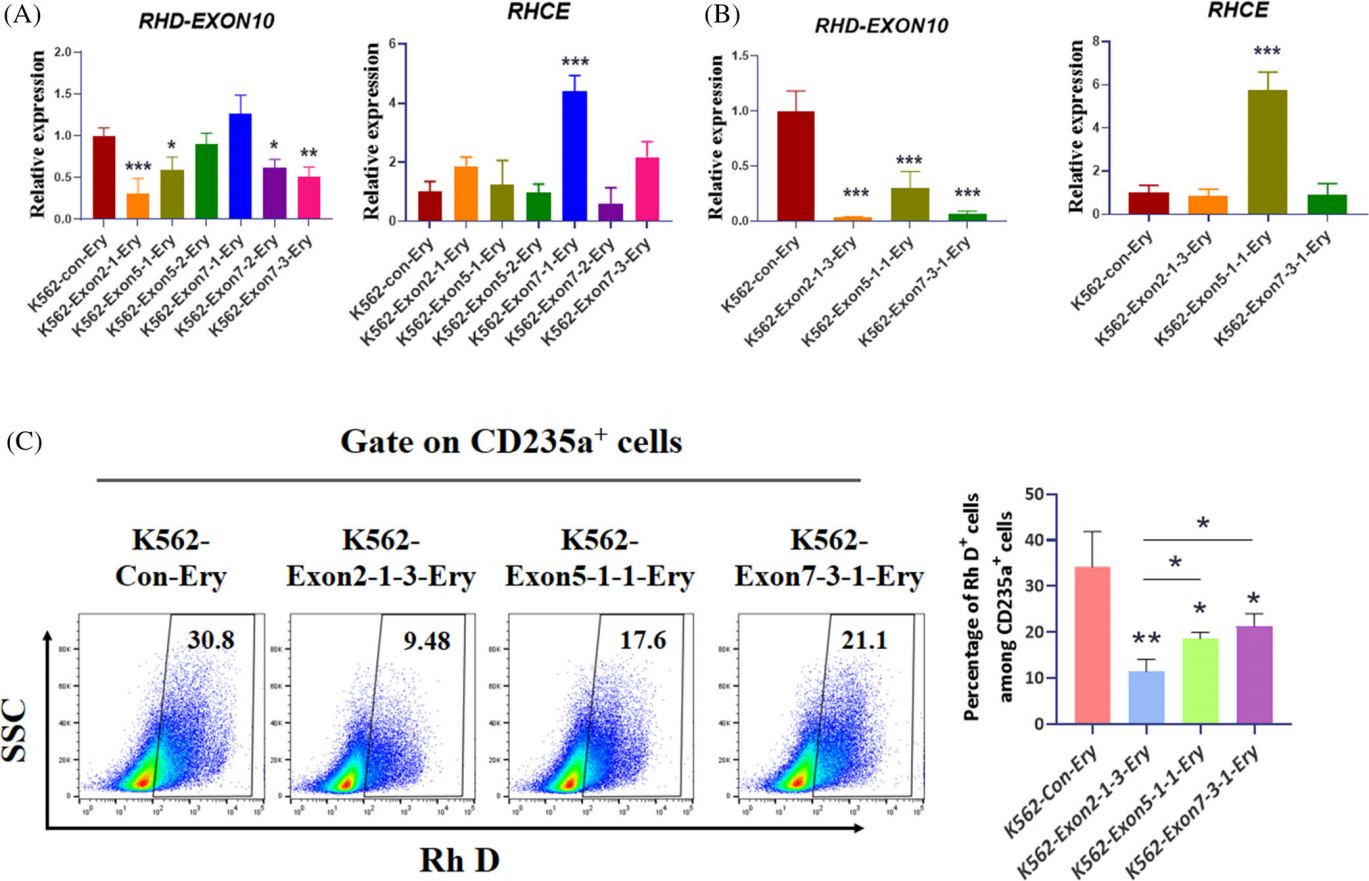
Selecting guide RNAs (gRNAs) targeting the human *RHD* gene. K562 cells stably transfected with LentiCRISPR v2‐gRNAs were analysed for the knockout effect on the *RHD* gene. **(**A) Quantitative reverse‐transcription‐polymerase chain reaction (qRT‐PCR) was performed to detect the expression of *RHD* and *RHCE* genes in K562‐con‐Ery, K562‐exon 2‐1‐Ery, K562‐exon 5‐1‐Ery, K562‐exon 5‐2‐Ery, K562‐exon 7‐1‐Ery, K562‐exon 7‐2‐Ery and K562‐exon 7‐3‐Ery cells. *RHD‐*exon 10 was designed to indicate the expression of the *RHD* gene using exon 10 qRT‐PCR. The results were obtained from three independent replicate experiments, with *GAPDH* used as the internal reference and the K562‐con‐Ery group set as 1. Results are expressed as mean ± standard deviation, *p < 0.05, **p < 0.01 and ***p < 0.001. (B and C) K562‐exon 2‐1‐Ery, K562‐exon 5‐1‐Ery and K562‐exon 7‐3‐Ery cells were subjected to single cell cloning, qRT‐PCR analysis of the expression of the *RHD* and *RHCE* genes (B), and FCM assay the expression of Rh D protein (C). Results were obtained from three independent replicate experiments and are expressed as mean ± standard deviation, *p < 0.05, **p < 0.01 and ***p < 0.001. Ery, Erythrocyte.

To validate the activity of the designed gRNAs, we transfected plasmids encoding both Cas9 and gRNA into K562 cells, selected puromycin, and then performed qRT‐PCR and flow cytometry (FCM) assays. qRT‐PCR showed that the gRNA of exon 2‐1, exon 5‐1 and exon 7‐3 significantly reduced *RHD* gene expression without off‐target effects on the *RHCE* gene (Figure 1A). These three groups of gRNA‐transfected K562 cells were subjected to single‐cell cloning, followed by RT‐qPCR and FCM assays. Finally, we determined that the gRNA targeting exon 2 was more efficient than the others (Figure 1B,C). gRNA of exon 2‐1 was selected for further experiments.

### 3.2 Generation of hiPSC clones containing *RHD* mutations

**FIGURE 2:**
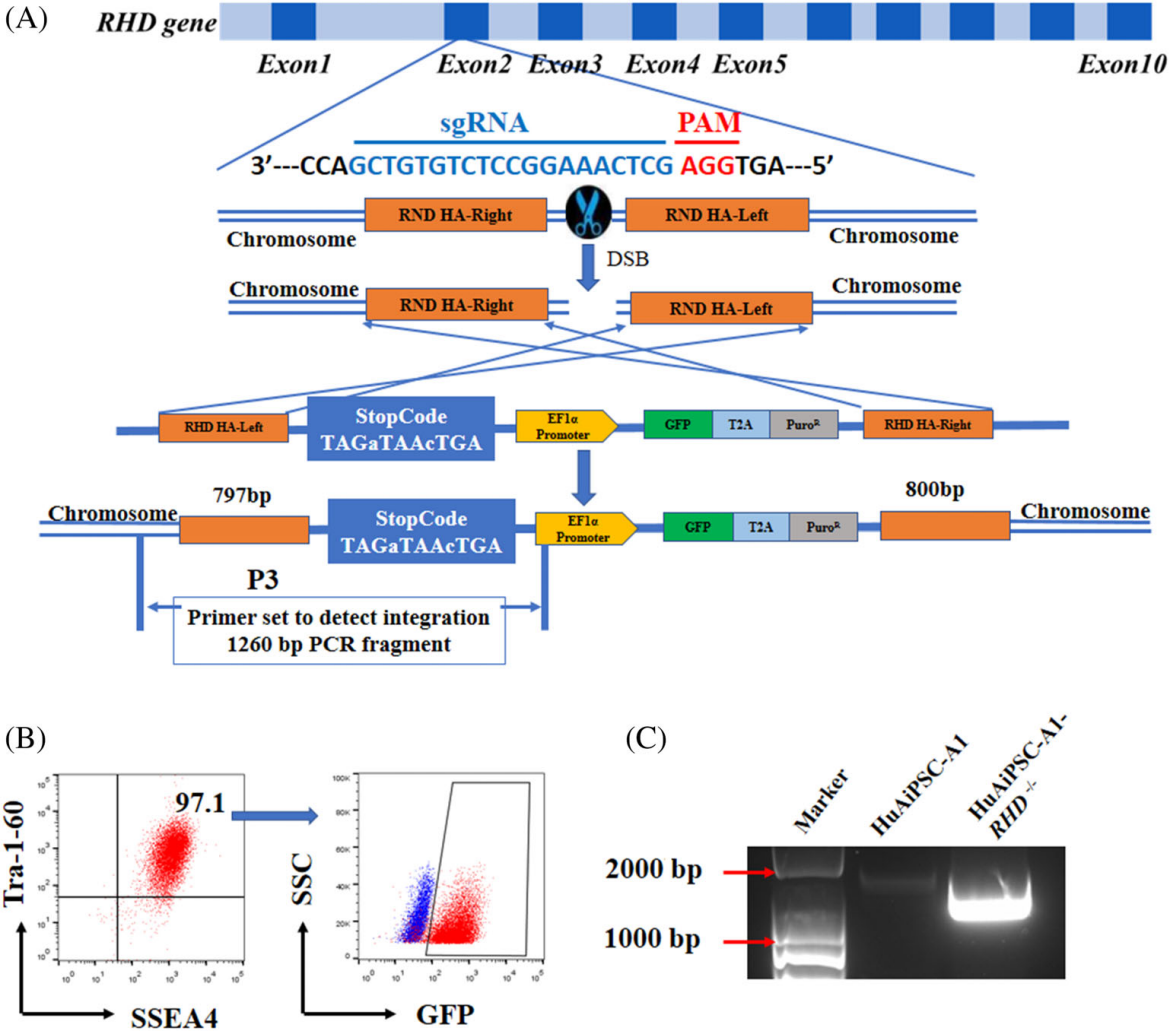
Generation of the *RHD* knockout human induced pluripotent stem cells (hiPSC) line. (A) Schematic overview of the gene targeting strategy to knockout *RHD* using the CRISPR/Cas9 system based on HDR. Guide RNA targeted to exon 2 was selected for *RHD* knockout. Dark blue boxes indicate exons. sgRNA target sites and PAM sequences are shown in blue and red letters, respectively. The donor construct comprised a strong stop code, an EF1α promoter, two screening tag (GFP and puromycin) and two homology arms. (B) FACS SSEA4^+^Tra‐1‐60^+^GFP^+^ cells. (C) Polymerase chain reaction (PCR) confirms the knockout of *RHD* in the HuAiPSC‐A1‐RHD^−/−^ cells. Properly targeted clones were validated using a PCR strategy with specific primer sets (P3) located in the right homology arm and stop codon, respectively.

A homology‐directed repair (HDR)‐based CRISPR/Cas9 system was used for *RHD* gene knockout in hiPSCs instead of the classical non‐homologous end‐joining (NHEJ)‐based CRISPR/Cas9 system. The homologous arms were designed to be identical to the side sequence around the exon 2‐1 gRNA target site of *RHD* gene loci, and a strong stop code together with the EF1α promoter driving GFP and puromycin was inserted to disrupt *RHD* expression (Figure 2A). After puromycin selection, followed by GFP‐positive sorting, potential candidates for mutant cell clones were confirmed using electrophoresis and sequencing (Figure 2B,C; Figure S2). The unique electrophoresis band and sequencing results suggested the homogeneity of the selected hiPSC clone. Consequently, we obtained a mutated clone with a premature stop code (TAGaTAAcTGA) in the *RHD* coding sequence (Figure S2).

### 3.3 Initial characterization of *RHD* knockout iPSC colonies

**FIGURE 3:**
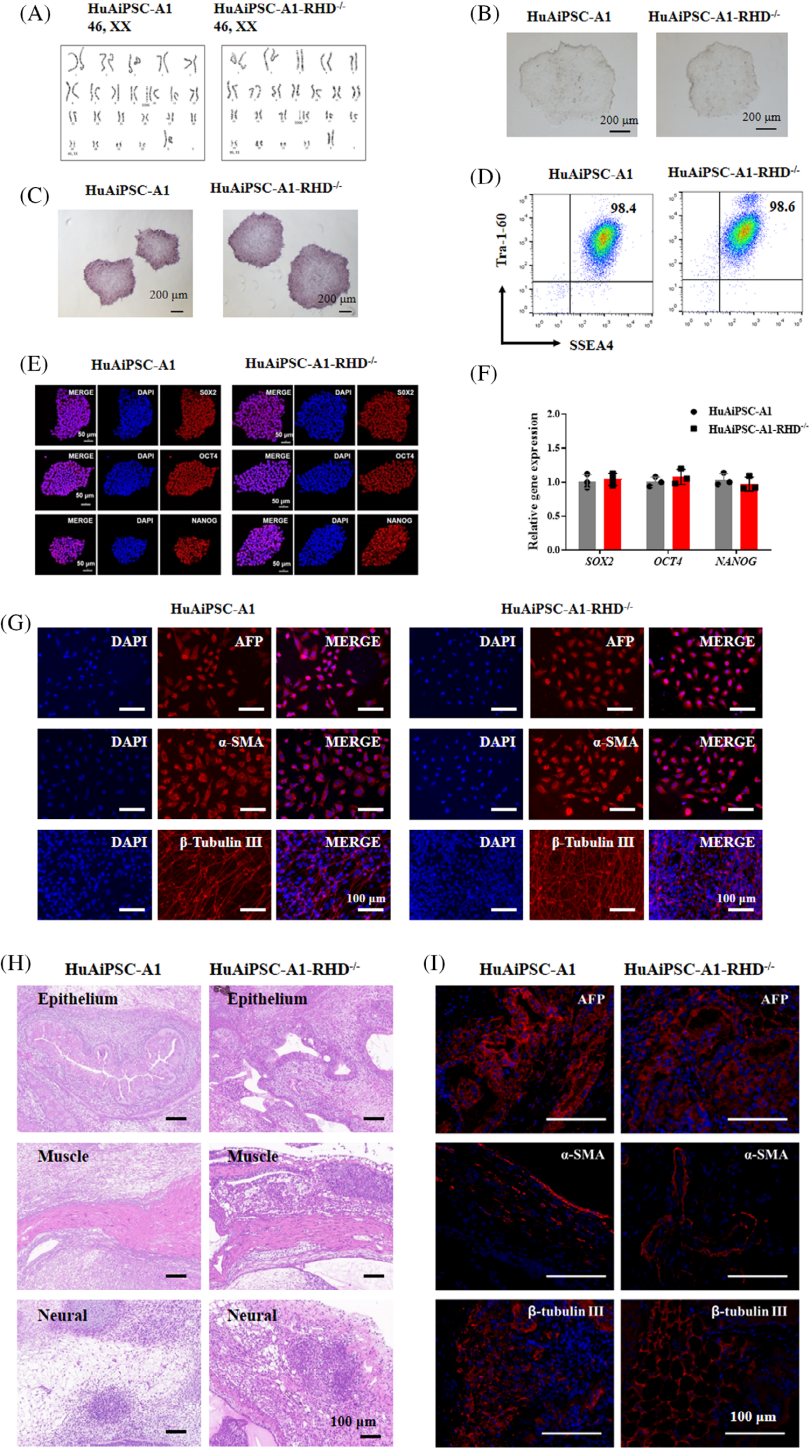
Characterization of the *RHD* knockout human induced pluripotent stem cells (hiPSC) line, HuAiPSC‐A1‐RHD^−/−^. (A). The HuAiPSC‐A1‐RHD^−/−^ cells show normal karyotype (46, XX). (B). HuAiPSC‐A1‐RHD^−/−^ cells exhibit normal morphology. (C–F) The HuAiPSC‐A1‐RHD^−/−^ cells exhibit positive alkaline phosphatase activity, express hiPSC surface markers (SSEA‐4 and Tra‐1‐60), hiPSC pluripotency factors (SOX2, OCT4 and NANOG), and hiPSC pluripotency genes (*SOX2*, *OCT4* and *NANOG*). PCR reactions were normalized to *GAPDH* and plotted relative to expression levels in HuAiPSC‐A1. Error bars indicate ± standard deviation of triplicates. n.s. indicates non‐statistically significant differences. (G–I) The HuAiPSC‐A1‐RHD^−/−^ cells maintained pluripotency. (G) Immunofluorescence staining revealed that the HuAiPSC‐A1‐RHD^−/−^ cells are capable of differentiating to the endodermal (alpha fetoprotein, AFP), mesodermal (alpha smooth muscle actin, α‐SMA) and ectodermal (β‐Tubulin III) lineages after EB induction in vitro. (H and I) To further evaluate the pluripotency of the HuAiPSC‐A1‐RHD^−/−^ cells, we performed in vivo teratoma formation assays. We injected HuAiPSC‐A1 and HuAiPSC‐A1‐RHD^−/−^ cells intramuscularly into NOD/SCID mice. Histological examination showed that the tumour contained tissues corresponding to the three embryonic germ layers, including epithelium (endoderm), muscle (mesoderm) and neural (ectoderm). Immunofluorescence staining revealed that tumour contains three germ tissue layers, including endodermal (AFP), mesodermal (α‐SMA) and ectodermal (β‐Tubulin III) lineages.

*RHD* knockout hiPSC colonies (HuAiPSC‐A1‐RHD^−/−^) showed a normal karyotype (46, XX) and no mycoplasma contamination (Figure 3A and S3). Short tandem repeat (STR) analysis confirmed that the hiPSC line shared a 100% matched genetic profile with wild‐type (WT) HuAiPSC‐A1 (Figure S4). HuAiPSC‐A1‐RHD^−/−^ cells exhibited a typical hiPSC‐like morphology and AP activity (Figure 3B,C). The pluripotency of HuAiPSC‐A1‐RHD^−/−^ cells was confirmed using FCM analysis of pluripotency markers (SSEA‐4 and TRA‐1–60), immunostaining for pluripotency markers (SOX2, OCT4 and NANOG), and qRT‐PCR for pluripotency genes (*SOX2*, *OCT4* and *NANOG*; Figure 3D–F). This cell line had the potential to differentiate into all three germ layers in vitro in the EB differentiation assays (Figure 3G) and in vivo in the teratoma formation assays (Figure 3H,I).

### 3.4 Optimized differentiation scheme towards erythroid lineage

**FIGURE 4:**
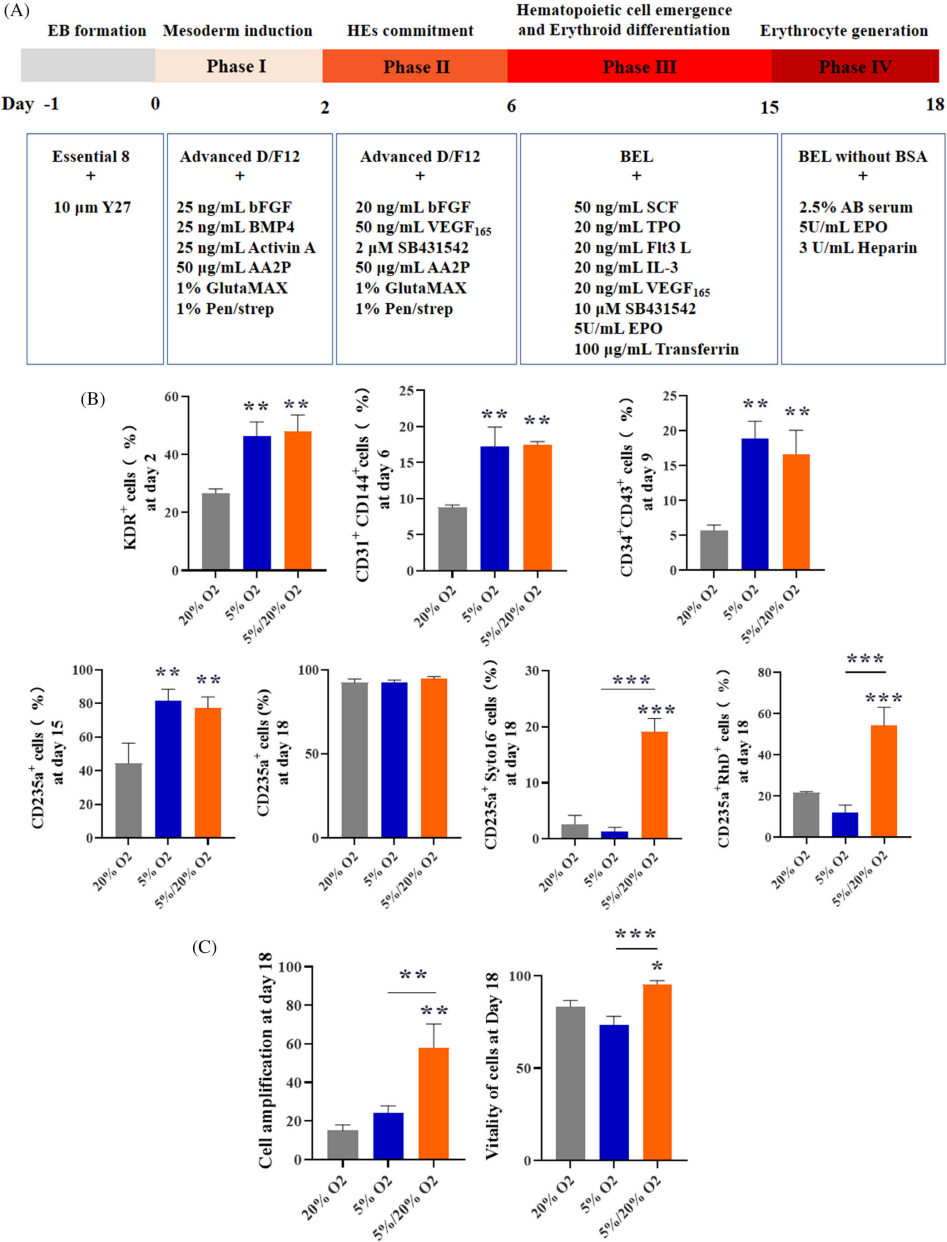
The efficient generation of erythroblasts from HuAiPSC‐A1‐RHD^−/−^ cells using a four‐step differentiation strategy. (A) Schematic diagram showing induction of human induced pluripotent stem cells (hiPSCs) to erythroid differentiation. (B) Flow cytometry analysis of expression of stage‐specific markers during induction of erythroid‐lineage in the 20% O_2_, 5% O_2_ and 5/20% O_2_ groups. Results were derived from three independent replicate experiments and are expressed as mean ± standard deviation, **p < 0.01 and ***p < 0.001. (C) Erythroid cell expansion fold and cell viability obtained in the 20% O_2_, 5% O_2_ and 5/20% O_2_ groups. Results were obtained from three independent replicate experiments and are expressed as mean ± standard deviation, *p < 0.05, **p < 0.01 and ***p < 0.001.

Studies have shown enhanced in vitro expansion of HSPCs and early development of erythroid clones when cultured under hypoxic conditions (1%–5% O_2_).^16^, ^17^, ^25^ However, hypoxia inhibits the terminal expansion and maturation of erythroid precursors.^18^ To obtain more mature erythrocytes with high CD235a expression, we set up an 18‐day stepwise induction protocol and analysed the influence of alterations in oxygen concentration during the process of induction (Figure 4A).

Three oxygen conditions, namely normoxia, hypoxia and hypoxia–normoxia culture conditions were established by setting the oxygen concentration at 20%, 5% and 5% for the first three phases and 20% for induction phase IV, respectively. By induction Day 18, the high levels of CD235a expression confirmed that these three conditions produced virtually pure populations of erythroid cells (Figure 4B). Significantly, we found that 5% oxygen followed by 20% oxygen resulted in more enucleated erythrocytes (CD235a^+^Syto16^−^) and higher expression of Rh D in CD235a^+^ cells than in cells under 20% oxygen or 5% oxygen (Figure 4B). The group treated with 5% oxygen followed by 20% oxygen had a higher HE marker (KDR/CD31) on Day 6 HSPC marker (CD34/CD43) on Day 9, and erythroid progenitor cell marker (CD71) on Day 15 than the other groups (Figure 4B). Moreover, the group treated with 5% oxygen followed by 20% oxygen had a higher expansion (measured by erythroid cells generated from one initially plated hiPSC) and cell vitality than the others (Figure 4C). Collectively, 5% oxygen followed by 20% oxygen resulted in higher haematopoietic and erythroid expansion efficiency as well as more mature erythrocytes compared with 5% and 20% oxygen.

### 3.5 Impact of *RHD* knockout on HuAiPSC‐A1 differentiation and function

**FIGURE 5:**
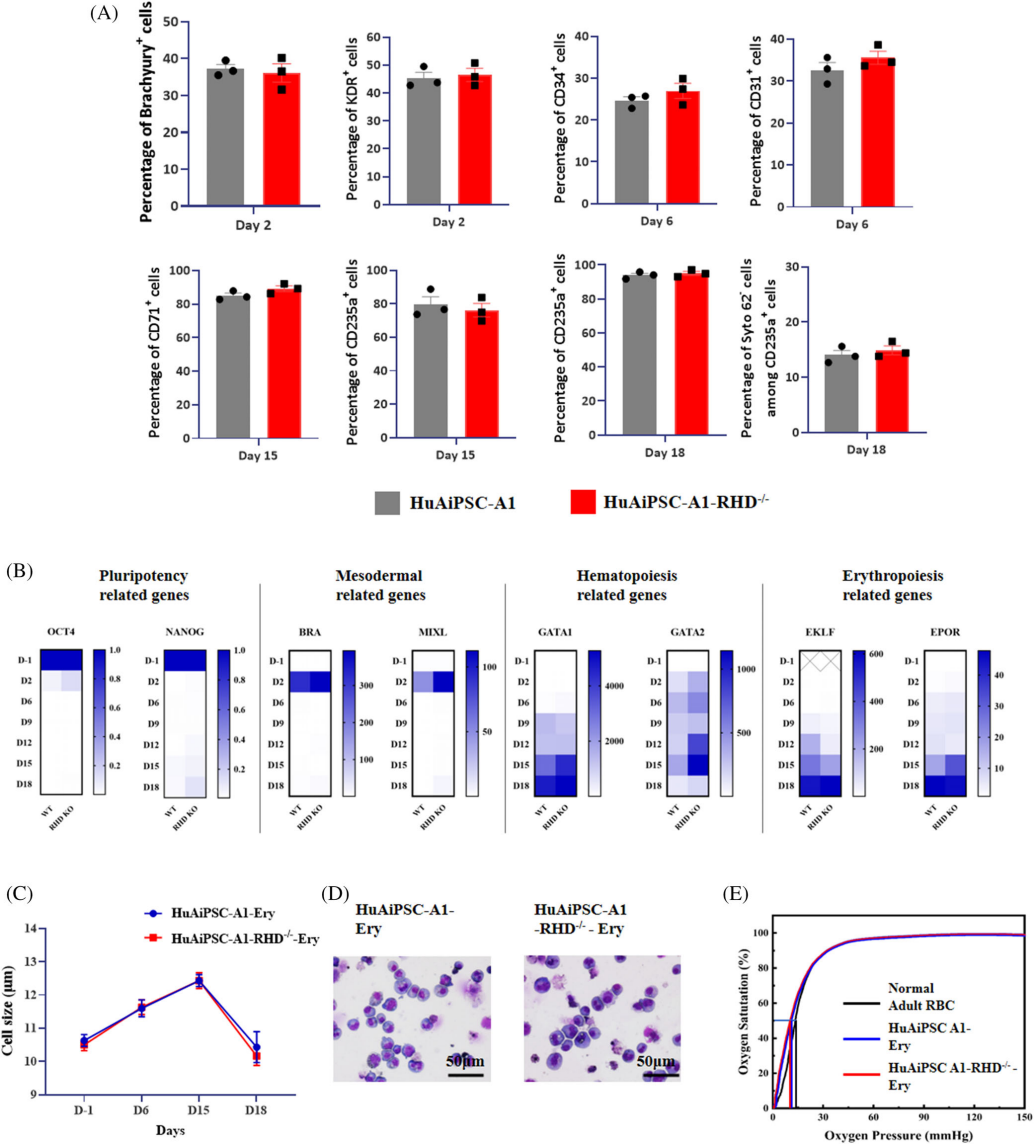
The *RHD* knockout does not affect the differentiation and function of HuAiPSC‐A1. (A, B) HuAiPSC‐A1 and HuAiPSC‐A1‐RHD^−/−^ cells were induced to differentiation for 18 days and subjected to FCM and qRT‐PCR analysis, at the indicated time point. (A) The expression of the PS (PS)/early mesoderm marker (Brachyury^+^ and KDR^+^) on Day 2, HE marker (CD31^+^) and early haematopoietic markers (CD34^+^) on Day 6, early erythroid progenitor marker (CD71^+^) and mature erythroid cell marker (CD235a^+^) on Day 15, and mature erythroid cell marker (CD235a^+^) and enucleated cell indicator (Syto62^−^) on Day 18 were tested using FCM. Results were obtained from three independent replicate experiments and are expressed as mean ± standard deviation, with n.s. indicating that the differences are not statistically significant. (B) The expression level of pluripotency genes (*OCT4* and *SOX2*), mesoderm genes (*BRA* and *MIXL*), haematopoietic related genes (*GATA1* and *RUNX1*) and erythroid related genes (*EKLF* and *EPOR*) were detected using qRT‐PCR. The results were derived from three independent replicate experiments with *GAPDH* as an internal reference and wild‐type group set as 1 for statistical analysis. (C–E) HuAiPSC‐A1 and HuAiPSC‐A1‐RHD^−/−^ cells were induced for differentiation for 18 days and performed to cell size (C), cell morphology (D) and oxygen‐carrying capacity (E) detection. Ery, Erythrocyte; RHD KO, HuAiPSC‐A1‐RHD^−/−^; WT, HuAiPSC‐A1.

We evaluated whether CRISPR/Cas9‐induced *RHD* knockout affects erythrocyte generation from iPSCs or oxygen‐carrying functions. FCM analysis showed that at each stage of differentiation induction, the expression levels of the primitive streak (PS)/early mesoderm (Brachyury and KDR), HE (CD31), early haematopoietic (CD34 and CD43), early erythroid progenitor (CD71), and mature erythroid cell markers (CD235a) did not change between *RHD* knockout (HuAiPSC‐A1‐RHD^−/−^) and WT hiPSC clones (HuAiPSC‐A1; Figure 5A). At the end of differentiation, for both cell types, more than 93% of the cells expressed erythrocyte marker CD235a, whereas the majority of the cells did not express megakaryocytic antigens, myelomonocytic antigens, and other haematopoietic progenitor antigens (Figure S5), corroborating that *RHD* knockout did not affect haematopoietic differentiation. Furthermore, qRT‐PCR analysis also indicated that the expression levels of pluripotency genes (*OCT4* and *SOX2*), mesoderm genes (*BRA* and *MIXL*), haematopoietic‐related genes (*GATA1* and *RUNX1*) and erythroid‐related genes (*EKLF* and *EPOR*) did not change between *RHD* knockout and WT hiPSC clones (Figure 5B), suggesting that CRISPR/Cas9‐induced *RHD* knockout does not affect the erythroid differentiation process. Moreover, the average cell size during differentiation and the cell morphology at the end of differentiation were identical between *RHD* knockout and WT iPSCs (Figure 5C,D), suggesting that *RHD* knockout does not affect differentiation. For functional comparison between erythrocytes generated from *RHD* knockout and WT hiPSCs, we measured their oxygen‐carrying capabilities. The oxygen binding and dissociation curves obtained were similar for both cell types (Figure 5E), indicating that CRISPR/Cas9‐induced *RHD* knockout did not affect RBC function. Collectively, these results indicate that erythrocyte differentiation and the oxygen‐carrying function of these derived erythrocytes are not affected by the CRISPR/Cas9‐induced *RHD* mutation in HuAiPSC‐A1.

### 3.6 Rh D antigen expression in HuAiPSC‐A1‐RHD ^−/−^ erythrocytes

**FIGURE 6:**
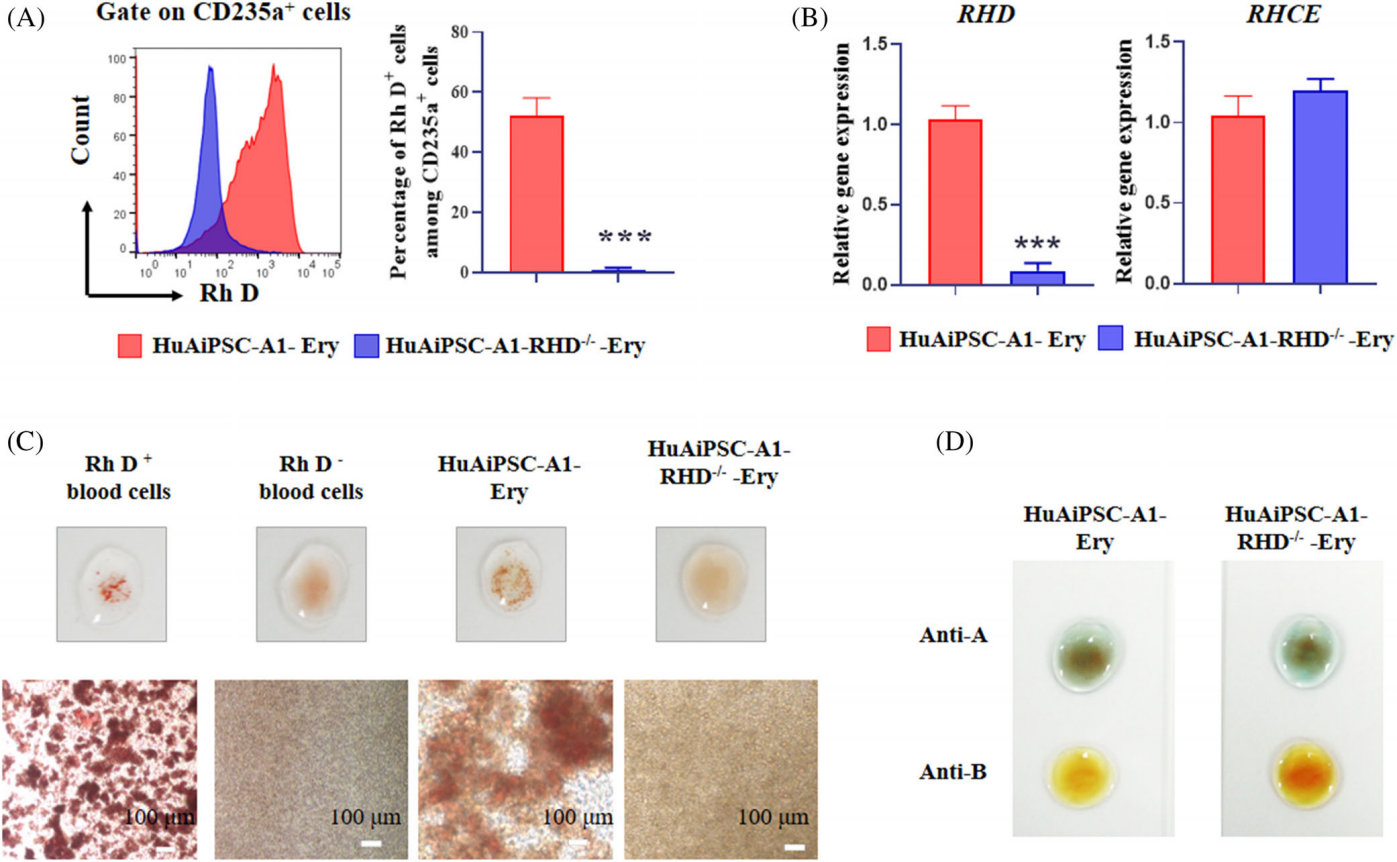
D antigen and *RHD* gene expression in HuAiPSC‐A1‐RHD^−/−^‐Erythrocytes. HuAiPSC‐A1 and HuAiPSC‐A1‐RHD^−/−^ were induced towards erythroid cells for 18 days. (A). FCM was used to analyse the percentage of Rh D antigen‐positive cells in the population of CD235a‐positive cells. Typical FCM diagrams (left) and data statistics (right) of three independent replicate experiments. Results are expressed as mean ± standard deviation, ***p < 0.001. (B) HuAiPSC‐A1‐ and HuAiPSC‐A1‐RHD^−/−^‐induced erythrocytes were analysed for the expression of *RHD* and *RHCE* genes using qRT‐PCR. Results were obtained from three independent replicate experiments, with *GAPDH* as an internal reference and the HuAiPSC‐A1‐Ery group set as 1 for statistical analysis, results are expressed as mean ± standard deviation, ***p < 0.001, and n.s. indicates that non‐statistically significant differences. (C). Absence of Rh D antigen‐mediated agglutination in erythrocytes derived from the *RHD* knockout hiPSC line HuAiPSC‐A1‐RHD^−/−^. HuAiPSC‐A1 and HuAiPSC‐A1‐RHD^−/−^ cells were induced to differentiation for 18 days and subjected to an agglutination test using anti‐Rh D blood grouping reagents in 96‐well plates and on glass slides. Rh D‐positive and Rh D‐negative human peripheral blood cells were used as the controls. A representative photograph and photomicrographs of each cell line are shown. Scale bar, 100 μm. (D). Absence of A or B antigen‐mediated agglutination in erythrocytes derived from HuAiPSC‐A1‐RHD^−/−^ cells. HuAiPSC‐A1 and HuAiPSC‐A1‐RHD^−/−^ cells were induced to differentiation for 18 days and subjected to an agglutination test using anti‐A or anti‐B blood grouping reagents. Ery, Erythrocyte.

The primary goal of this study was to generate Rh‐D‐negative RBCs from genetically modified hiPSCs. Therefore, we evaluated Rh D antigen expression in erythrocytes generated from HuAiPSC‐A1 and HuAiPSC‐A1‐RHD^−/−^ clones using FCM. A previous study has shown that FCM is a sensitive way to detect Rh D antigen expression with a nonspecific background signal of approximately 1%.^7^ In our study, among the CD235a^+^ cells, the Rh D antigen expression rate was 52.17 ± 5.914% and 0.907 ± 0.668% in HuAiPSC‐A1‐Ery and HuAiPSC‐A1‐RHD^−/−^‐Ery cells, respectively (Figure 6A). The Rh D antigen of *RHD* knockout clones was identical to the nonspecific background level, suggesting the absence of Rh D antigen expression in erythrocytes generated from HuAiPSC‐A1‐RHD^−/−^ clones. *RHD* gene knockout was also confirmed at the mRNA level, and compared to HuAiPSC‐A1‐Ery, the expression of the *RHD* gene was significantly reduced in HuAiPSC‐A1‐RHD^−/−^‐Ery (Figure 6B).

*RHCE* genes are highly homologous with *RHD*, and previous studies have commonly reported off‐target effects on *RHCE* when *RHD* was targeted.^7^, ^26^ Next, we investigated whether the *RHD* knockout clones contained off‐target mutations arising from gene editing. No significant difference was detected in the expression of *RHCE* genes between HuAiPSC‐A1‐Ery and HuAiPSC‐A1‐RHD^−/−^‐cells through qRT‐PCR analysis (Figure 6B).

The blood type of the ‘universal’ RBCs derived from *RHD* gene knockout hiPSCs was finally verified using an agglutination test. HuAiPSC‐A1 and HuAiPSC‐A1‐RHD^−/−^ cells were induced for erythroid differentiation for 18 days using the optimized protocol, and blood group serological tests were performed. We used Rh D‐positive and Rh D‐negative blood cells as positive and negative controls, which showed agglutination and no agglutination, respectively. HuAiPSC‐A1‐RHD^−/−^‐Ery cells did not agglutinate with Rh D antiserum, demonstrating that they were Rh D‐negative cells (Figure 6C). The induced RBCs were tested as type O blood samples (Figure 6D).

## 4 DISCUSSION

The generation of universal RBCs to meet the increasing demand for blood donation has become the focus of researchers worldwide. Some reports have shown the possibility of producing seed cells (stem cells) of O‐type Rh D‐negative RBCs.^6^, ^8^, ^27^, ^28^ A pioneering study is *RHD* knockout from HiDEP‐1 immortalized erythroid progenitors with transcription activator‐like effector nucleases (TALEN).^7^ Later on, new strategies came out as multiple knockouts of rare blood type antigens from BEL‐A or hiPSCs, and *RHAG* knockout was taken instead of *RHD* knockout to derive a Rh_null_ phenotype.^29^, ^30^ Besides, a Rh_null_ hiPSC line was chosen for the creation of universal RBCs.^31^ However, O‐type Rh‐D‐negative RBCs have not yet been successfully identified. In this study, we took advantage of some pioneering techniques and veritably made O‐type Rh D‐negative RBCs, which may be useful for clinical applications in the future.

The selection of efficient seed cells is one of the key steps in producing RBCs. Although haematopoietic stem cells (HSCs) from cord blood, bone marrow or peripheral blood, pluripotent stem cells (PSCs), including embryonic stem cells and iPSCs, and immortalized erythroid progenitors have all shown the potential to be induced into RBCs in vitro, there are still shortcomings that remain to be overcome before clinical translation.^32^ HSCs are difficult to maintain in vitro and to be genetically manipulated.^33^ To induce PSCs towards RBCs used to be low efficient and low maturity.^34^ RBCs generated from immortalized erythroid progenitors typically show problems in enucleation and deformability.^34^, ^35^, ^36^ The karyotype abnormal was also reported in the immortalized BEL‐A line.^37^ Besides, mature RBCs can also be converted into universal RBCs by antigen mask, but the safety and in vivo function of these modified RBCs remained to be verified.^38^ We have currently discovered a highly haematopoietic differentiation preference hiPSCs with parent cells that were HuAECs.^19^ These cells have shown priority in efficient amplification and high haematopoietic differentiation performance. Thus, O‐type Rh D‐negative HuAiPSCs are superior seed cells for universal RBCs. Moreover, the optimized iPSC‐RBC induction protocol, which could result in approximately 94% CD235a^+^ erythrocytes and approximately 20% enucleated cells after 18 days of culture, also provided the possibility of efficiently generating blood type‐defined RBCs from these hiPSCs in vitro.

The pioneering study by Kim et al. suggested the construction of Rh D‐negative progenitor lines using the TALEN technique. However, it is complicated and cannot avoid off‐target effects, especially for *RHCE*, that is highly homologous with *RHD*.^7^ Here, we used the CRISPR/Cas9 technique and set up *RHD* knockout hiPSCs. The selection of the HDR‐based CRISPR/Cas9 system prevented the random modification of genomic DNA and subsequent heterogeneous clone formation, which may come from NHEJ‐based CRISPR/Cas9 editing.^39^ Considering future clinical translation of these iPSC‐induced RBCs, heterogeneous seed cells may come out of RBCs comprising both Rh D positive and negative cells, resulting in problems with transfusion. Single‐cell cloning is required but is difficult to achieve when the NHEJ‐based CRISPR/Cas9 system is chosen. Cell‐to‐cell contact is necessary for the stemness of human PSCs.^40^, ^41^ The HDR‐based CRISPR/Cas9 system unified the output of gene editing into one result, which was also confirmed through our sequencing results. The CRISPR/Cas9 based *RHD* knockout could be further improved by deleting exogenous selection markers when the gene editing procedure has been completed.^42^ To deplete *RHD* expression, some recent studies chose *RHAG*, which is the processor of *RHD* and *RHCE*, as target gene, or directly took *RHD* and *RHCE* double mutant hiPSCs (Rh_null_) as seed cells.^29^, ^30^, ^31^ However, the deletion of *RHAG* or Rh_null_ phenotype would result in membrane deformability and gas transport function defection, subsequently haematological abnormal.^26^, ^43^, ^44^ Considering Rh_null_ phenotype occurs in only 1 from 600,000 peoples,^26^ *RHD* specific knockout might be more applicable in clinical transfusion, meanwhile perform better in functional RBC derivation in vitro.

The third improvement in making universal RBCs was the optimization of the iPSC‐RBC induction protocol with an optimized oxygen concentration. Stepwise induction schemes include mesoderm patterning, haematopoietic progenitor induction, erythroid commitment and terminal maturation. We set the first three phases under hypoxic conditions, but the final phase under normoxic conditions, simulating the microenvironment of haematopoiesis and erythropoiesis. There have been reports suggesting a positive effect of hypoxia (1%–5% O_2_) on the expansion of HSPCs and a negative effect on the maturation of erythrocytes.^16^, ^17^, ^18^ Our results indicated that the first three stages of 5% oxygen followed by 20% oxygen for the final stage resulted in a higher erythroid expansion efficiency and even more mature erythrocytes. However, although most of the studies did not report the expression of *RHD* in iPSC‐derived RBCs, the expression of other blood group antigens was not available, which suggested that these induced RBCs were immature. Complete Rh D expression was reported only in fully mature RBCs.^45^ We are not sure whether these blood group antigens will appear in induced RBCs in vivo after transfusion. Using our optimized protocol, *RHD* was successfully expressed in the control group, suggesting the maturity of the induced RBCs.

In conclusion, using haematopoietic preference iPSCs, the HDR‐based CRISPR/Cas9 gene‐editing system, and an optimized induction protocol, we generated O‐type Rh D‐negative universal RBCs from *RHD* knockout HuAiPSCs. Its production is highly efficient, and the product shows great potential for clinical applications.

## AUTHOR CONTRIBUTIONS

Xuetao Pei and Xiaoyan Xie performed study concept and design; Lei Xu, Quan Zeng, Liqing Liang, Zhou Yang, Mingyi Qu, Xin Yuan, Lin Chen and Zeng Fan provided acquisition, analysis and interpretation of data, and statistical analysis; Lei Xu, Jing Zhang and Xiaoyan Xie wrote and edited the manuscript; Huilin Li, Bowen Zhang, Wen Yue, Lijuan He and Xue Nan provided technical and material support. All authors read and approved the final paper.

## FUNDING INFORMATION

This work was supported by the National Key Research and Development Program of China (2017YFA0103100, 2017YFA0103103, 2017YFA0103104), National Nature Science Foundation of China (No. 32200589), The Guangzhou Scientific Research Program (No. 201904010378), Science and Technology Program of Guangzhou, China (No. 202002030025).

## CONFLICT OF INTEREST STATEMENT

The authors declare no conflict of interest.

## Supporting information


**Data S1.** Supporting information.

## Data Availability

All data needed to evaluate the conclusions in the paper are present in the paper and/or the Supplementary Materials. Additional data related to this paper may be requested from the authors.
