# Supplementary material for: Generation of Rh D‐negative blood using CRISPR/Cas9
Source: Cell Prolif. 2023 Apr 25;56(11):e13486. doi: 10.1111/cpr.13486 (PMC10623963; doi:10.1111/cpr.13486)
Supplement: Supplementary file 1 — Data S1. Supporting information. [file CPR-56-e13486-s001.docx]

**Supplementary Figures**


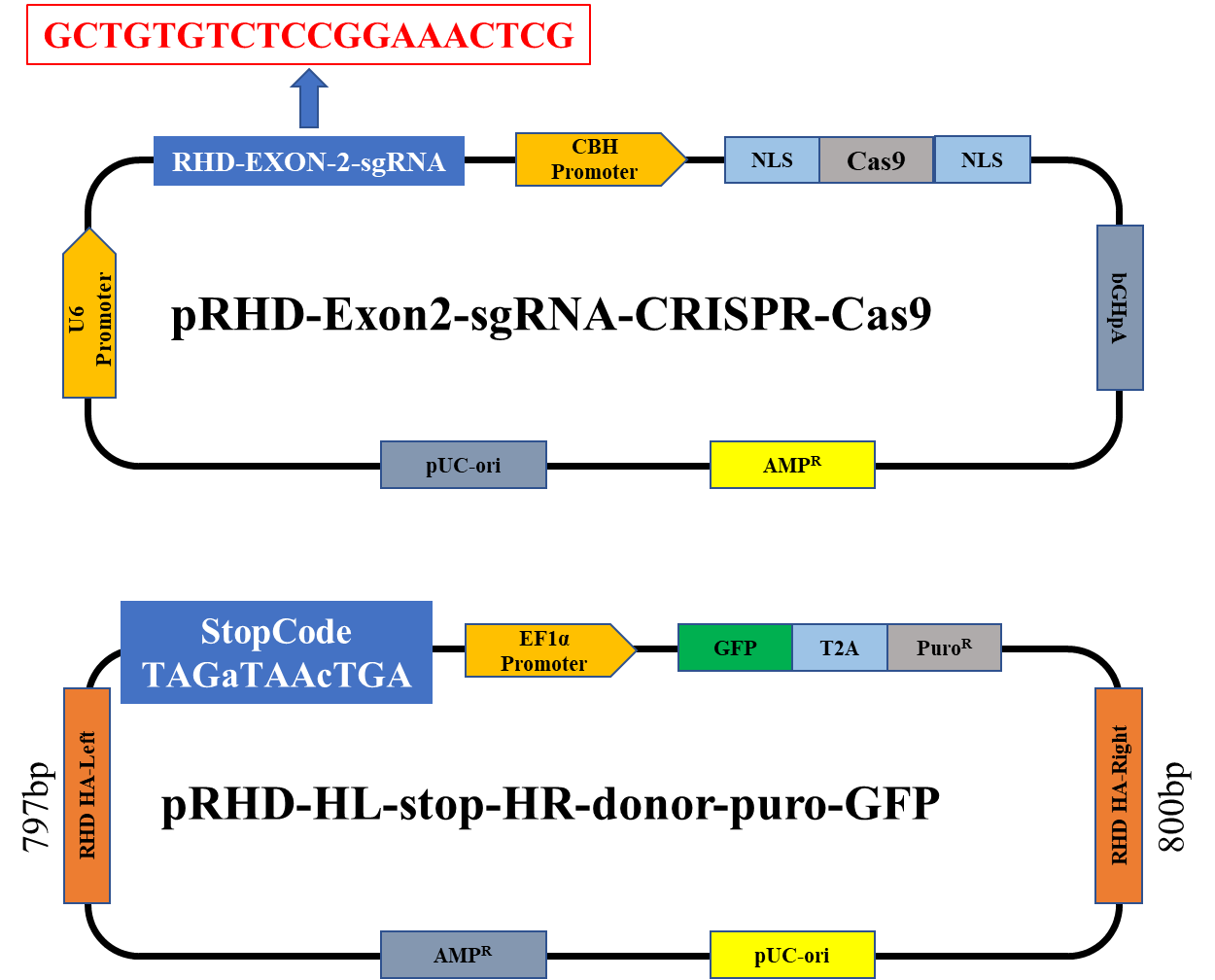


**Figure S1. The CRISPR/Cas9 vectors targeting RHD gene.**

Structures of pRHD-Exon2-sgRNA-CRISPR/Cas9 and homologous recombination template vector pRHD-HL-stop-HR-donor-puro-GFP vectors.


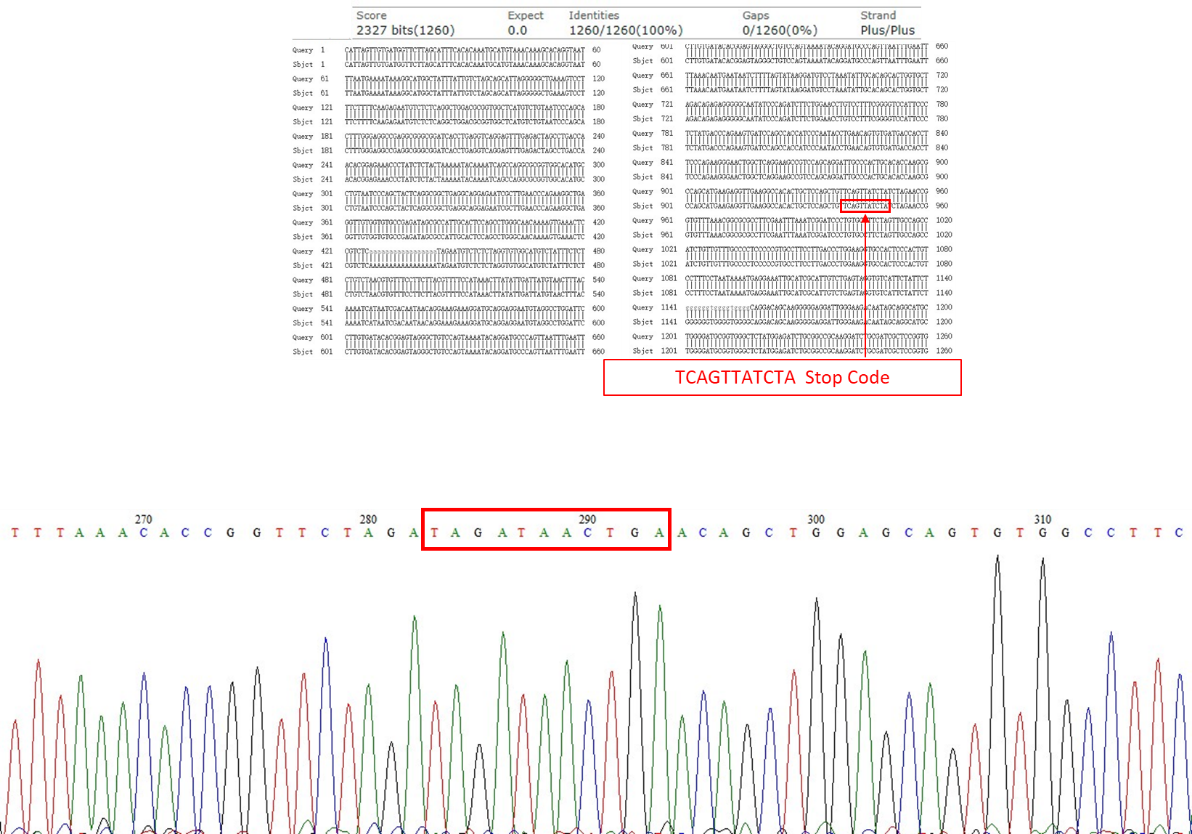


**Figure S2.** **DNA sequences of PCR amplified RHD exon 2 loci of HuAiPSC-A1-RHD ^-/-^ .**

Sequencing results indicated the HuAiPSC-A1-RHD^-/-^ clone conceived premature stop code (TAGaTAAcTGA) in *RHD* exon 2.


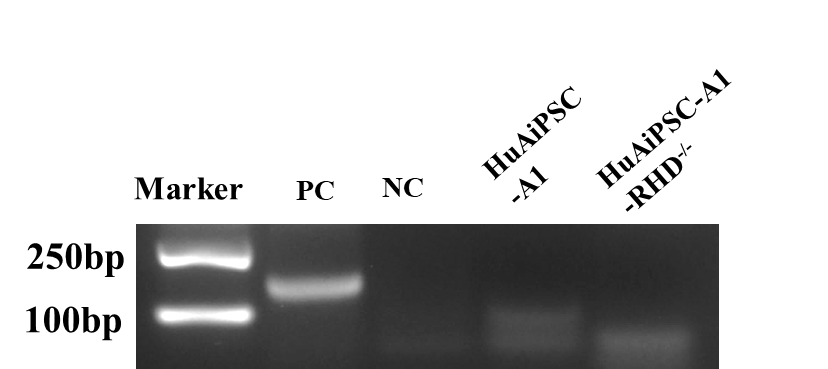


**Figure S3.** **Mycoplasma test of HuAiPSC-A1-RHD^-/-^** .

PCR identified that HuAiPSC-A1-RHD^-/-^ cells were free of mycoplasma contamination.


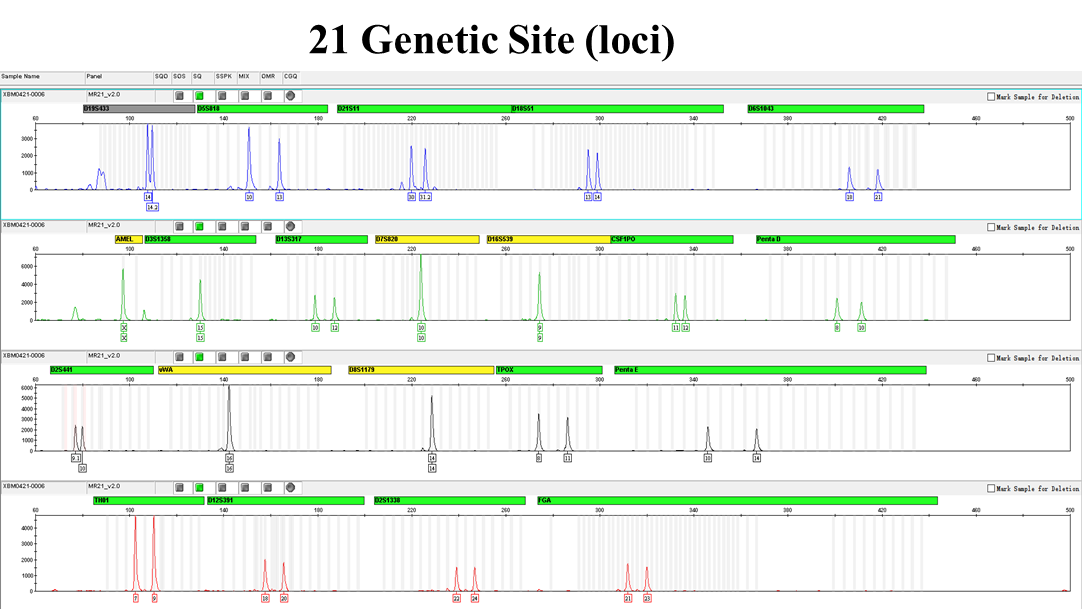


**Figure S4. STR analysis of HuAiPSC-A1-RHD^-/-^**

Short tandem repeat (STR) analysis confirmed that HuAiPSC-A1-RHD^-/-^ matched 100% with wild-type HuAiPSC-A1 in gene profile.


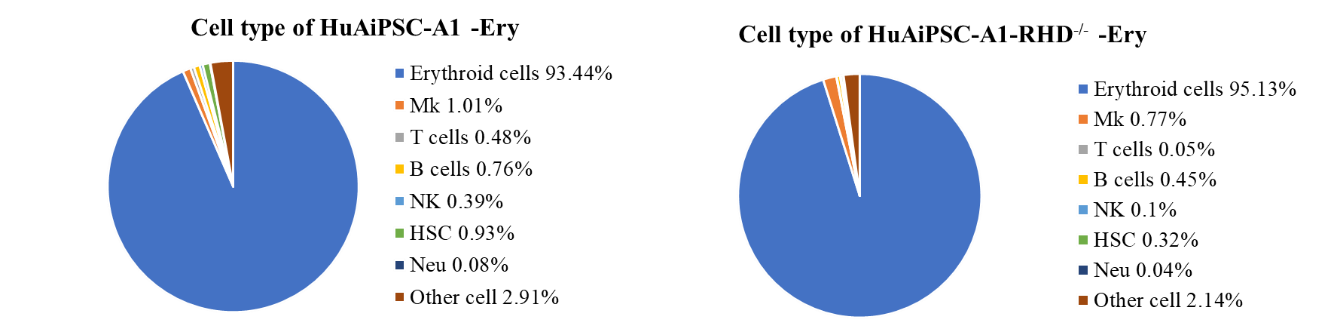


**Figure S5. Cell contents of HuAiPSC-A1-RHD^-/-^ derivations from erythroid induction day 18.**

HuAiPSC-A1 and HuAiPSC-A1-RHD^-/-^ cells were induced to differentiation for 18 days and subjected to the analysis of the percentage of CD235a^+^ erythrocytes, CD14^+^ macrophages, CD3^+^ T lymphocytes, CD19^+^ B cells, CD56^+^ NK cells, CD34^+^CD90^+^ hematopoietic stem cells, CD66b^+^ neutrophils by FCM. Abbreviations: Mk. Macrophages, Neu: Neutrophils, Ery: Erythrocyte.

**Table S1. Primer**

| **Gene Name** | **Forward** | **Reverse** |
| --- | --- | --- |
| RHD-Left-homo | ggtacc tggacgcggtggctcatgt | tctagaTAGaTAAcTGAacagctggagcagtgtggcc |
| RHD-Right-homo | gttaac gtctccggaaactcgaggtgagga | GTCGACcctttttcaaaactggaattaaac |
| RHD-genome | caccggagcgatcgcagatcctt | cattagttgtgatggttcttagcat |
| P3 | CACCGGAGCGATCGCAGATCCTT | CATTAGTTGTGATGGTTCTTAGCAT |
| *GAPDH* | GAGTCAACGGATTTGGTCGT | TTGATTTTGGAGGGATCTCG |
| *OCT4* | CCTCACTTCACTGCACTGTA | CAGGTTTTCTTTCCCTAGCT |
| *SOX2* | CCCAGCAGACTTCACATGT | CCTCCCATTTCCCTCGTTTT |
| *NANOG* | ACAACTGGCCGAAGAATAGCA | GGTTCCCAGTCGGGTTCAC |
| *BRA* | TATGAGCCTCGAATCCACATAGT | CCTCGTTCTGATAAGCAGTCAC |
| *MIXL1* | GGCGTCAGAGTGGGAAATCC | GGCAGGCAGTTCACATCTACC |
| *GATA1* | CTGTCCCCAATAGTGCTTATGG | GAATAGGCTGCTGAATTGAGGG |
| *GATA2* | GCAACCCCTACTATGCCAACC | CAGTGGCGTCTTGGAGAAG |
| *EPOR* | TGGAGGACTTGGTGTGTTTCT | GCAACTCTAGGGGCACGAA |
| *EKLF* | GGTTGCGGCAAGAGCTACA | GTCAGAGCGCGAAAAAGCAC |
| *RHE* | TGAGGCACTTCTACGTGTTCG | CCACACTGACTGCTAGAGCATAG |
| *RHD-EXON10* | CCTCTCACTGTTGCCTGCATT | AGTGCCTGCGCGAACATT |

**Table S2. Key Resources Table**

| **Antibodies** | **Source** | **Identifier** |
| --- | --- | --- |
| Anti-Brachyury-PE | R&D | Cat#IC2085P |
| Anti-KDR-APC | R&D | Cat#FAB357A-100 |
| anti-SSEA-4 -AF 647 | BD | 560796 |
| Anti-CD34-APC | BD | Cat#555824 |
| Anti-CD45-PE | BD | Cat#555483 |
| Anti-CD43-BV421 | BD | Cat#562916 |
| Anti-CD71-APC | BD | Cat#551374 |
| Anti-CD235a-BV421 | BD | Cat#562938 |
| Anti-CD31-APC | Invitrogen | Cat#17-0319-42 |
| Anti-CD71-PECY7 | Invitrogen | Cat#25-0719-42 |
| SYTO 62 -APC | Invitrogen | Cat#S11344 |
| Anti-TRA-1-60-PE | MACS | Cat#130-122-921 |
| Rabit Anti-RHD | Biorbyt | Cat#orb312955 |
| Rabit anti-OCT4 | Abcam | Cat#ab181557 |
| Rabit anti-SOX2 | Abcam | Cat#ab92494 |
| Rabit anti-NANOG | Abcam | Cat#ab21624 |
| Anti-AFP antibody | Proteintech | Cat#14550-1-AP |
| Anti-α- SMA antibody | CST | Cat# 19245S |
| Anti-β-Tubulin III antibody | Sigma | Cat# T8660 |
|  |  |  |
| **Cytokines and Chemicals** | **Source** | **Identifier** |
| Activin A | R&D | Cat#338-AC |
| BMP-4 | R&D | Cat#314-BP |
| VEGF_165_ | R&D | Cat#293-VE |
| bFGF | Peprotech | Cat#100-18B |
| SCF | Peprotech | Cat#300-07 |
| Flt-3L | Peprotech | Cat#300-19 |
| IL-3 | Peprotech | Cat#200-03 |
| TPO | Peprotech | Cat#300-18 |
| EPO | Peprotech | Cat#100-64 |
| Transferrin | Sigma | Cat# T0665 |
| SB431542 | Selleck | Cat#S1067 |
| CHIR99021 | Selleck | Cat#S2924 |
| Y27632 | Selleck | Cat#S1049 |
| Heparin Solution | Stemcell | Cat# 07980 |
| Matrigel | Corning | Cat#354277 |
| Glutamax | Gibco | Cat#35050-061 |
| collagense D | sigma | Cat#11088866001 |
| Dnaes Ⅰ | sigma | Cat#11284932001 |
|  |  |  |
| **Culture Medium** | **Source** | **Identifier** |
| Essential 8 | Gibco | Cat# A1517001 |
| Advanced D/F12 | Gibco | Cat#12634-010 |
| IMDM | Gibco | Cat#31980-030 |
| F12 | Gibco | Cat#31765-035 |

**Table S3. The composition of BEL medium**

| **Media Component** | **Component Content** |
| --- | --- |
| IMDM/F-12 | 1:1 |
| Deionized BSA | 2.5 mg/mL |
| Synthechol Solution | 0.2 % |
| Linoleic Acid | 100 ng/mL |
| Linolenic Acid | 100 ng/mL |
| Ascorbic Acid 2-phosphate | 50 μg/mL |
| α-MTG | 450 μM |
| Glutamax | 2 mM |
| Protein-Free Hybridoma Mix | 5 % |
| Insulin-Transferrin-Selenium | 1 % |
